# Cognitive Performance and Biomarker Profiles in Puipui Malu Manatu (*Preserving Memory*) ‐ a Population‐Based Prevalence Study ADRD in American Samoa

**DOI:** 10.1002/alz70856_100469

**Published:** 2025-12-25

**Authors:** James E Galvin, Tulimalefo’i Vaofanua, Grace Grace Tuato’o, Toaiva Hall, Mahesh S Joshi, Marcia I Walker, Conor B Galvin, Vaatausili Tofaeono

**Affiliations:** ^1^ University of Miami Miller School of Medicine, Boca Raton, FL, USA; ^2^ American Samoa Community Cancer Coalition, Pago Pago, American Samoa, American Samoa; ^3^ American Samoa Community Cancer Coalition, Pago Pago, American Samoa

## Abstract

**Background:**

Puipui Malu Manatu is a NIA‐funded study of ADRD prevalence, risk factors, and biomarker profiles in American Samoa. We report cognitive and biomarker findings on the first 400 participants. Data collection is ongoing.

**Method:**

A probability sample of 981 Samoan elders age 50+ will be enrolled to (a) study ADRD health literacy, risk and resilience factors, and cognitive screening tools, (b) have a Gold Standard evaluation using the UDSv3.0, and (c) explore genetic and blood‐based biomarkers including PrecivityAD2 (C2N Diagnostics).

**Result:**

The cohort had a mean age of 60.4 ± 7.3y, 11.3 ± 2.5y of education, and 58% women. The mean MoCA score was 14.3 ± 3.7 (Range 3‐22) and the mean Cognivue *Clarity* score was 54.8 ± 20.2 (Range 0‐90). The mean QDRS score was 1.9 ± 2.4 (Range 0‐15.5). The cohort reported 39% subjective cognitive decline, and more than half endorsed mood disturbance. The mean CDR‐SB was 0.5 ± 1.0 (Range 0‐10) with 69% CDR 0, and 31% CDR 0.5+. Cognitively impaired participants were older, male, had more medical co‐morbidities, more subjective complaints, and lower MoCA scores. There were no differences between groups in the UDSv3.0 neuropsychological battery except for verbal fluency. On average, Samoan elders scored >2 SD below normative values on all cognitive tests. ApoE ε3/ε3 was the most genotype (49%) with 33% ApoE ε4 carriers equally distributed between CDR 0 and CDR 0.5+. 19% had low Aβ42/40 ratios and 44% had elevated pTau217% ratios but there were no differences in AD biomarkers between CDR 0 and CDR 0.5+. Aβ42/40 ratio was weakly correlated with vascular risk factors, depression, and cognitive performance, while pTau217% ratios and APS2 scores were moderately correlated with age and CDR‐SB scores.

**Conclusion:**

The prevalence of cognitive impairment in American Samoa is 31%. Most cases of cognitive impairment appear not to be related to AD pathology and ApoE ε4 does not appear to be a risk factor. Our study findings suggest that (a) exploration of non‐AD biomarkers is needed, (b) cultural tailoring and re‐examination of inter‐individual normative values for cognitive tests are important, and (c) intra‐individual change as measured by the CDR may be a more valid method of dementia detection.

